# Hepatocellular Neoplasm of Uncertain Potential of Malignancy or Well-Differentiated Hepatocellular Carcinoma Arising within Hepatocellular Adenoma

**DOI:** 10.1155/2023/2831510

**Published:** 2023-03-29

**Authors:** Elisabete Campos, Roberto Silva, Sara Rodrigues, Marinho de Almeida, Joanne Lopes, Marisa Aral, Humberto Cristino, Luís Graça, Elisabete Barbosa

**Affiliations:** ^1^Hepato-Bilio-Pancreatic and Splenic Unit, General Surgery Department, São João University Medical Center, Oporto, Portugal; ^2^Pathology Department, São João University Medical Center, Oporto, Portugal

## Abstract

**Background:**

Hepatocellular adenoma (HCA) is an uncommon solid, solitary, benign liver lesion that develops in an otherwise normal-appearing liver. Hemorrhage and malignant transformation are the most important complications. Risk factors for malignant transformation include advanced age, male gender, use of anabolic steroids, metabolic syndrome, larger lesions, and beta-catenin activation subtype. The identification of higher risk adenomas enables the selection of patients most suitable for aggressive treatment and those who benefit with surveillance, minimizing the risks for these predominantly young patients. *Case Presentation*. We present the case of a 29-year-old woman with a history of oral contraceptive intake for 13 years, which was sent to evaluation in our Hepato-Bilio-Pancreatic and Splenic Unit because of a large nodular lesion in segment 5 of the liver, compatible with HCA, and was proposed to surgical resection. Histological and immunohistochemical investigation revealed an area with atypical characteristics, suggesting malignant transformation.

**Conclusions:**

HCAs share similar imaging characteristics and histopathological features with hepatocellular carcinomas; therefore, immunohistochemical and genetic studies assumes great importance to discriminate adenomas with malignant transformation. Beta-catenin, glutamine synthetase, glypican-3, and heat-shock protein 70 are promising markers to identify higher risk adenomas.

## 1. Introduction

Hepatocellular adenoma (HCA) is a rare solid, solitary, benign liver lesion that develops in an otherwise normal-appearing liver [1–4]. Often, they are found in young women in association with use of estrogen-containing medications. The annual incidence of HCA in users of oral contraceptives is approximately 30–40 cases per million in comparison with one case per million for non-users [2, 3].

The most important complications of HCA are hemorrhage (25–64%) and malignant transformation into hepatocellular carcinoma (HCC; 4–10%), which are the two main reasons for surgical treatment. Risk factors for malignant transformation include advanced age, male gender, use of anabolic steroids, metabolic syndrome, lesion size >5 cm, and beta-catenin activation subtype [2, 4–6].

In women the size of HCA has, by current consensus, remained the main decision criterion in determining whether or not resection is indicated, based upon the observation that intratumoral bleeding and malignant transformation only seldom takes place in lesions smaller than 5 cm [5, 7]. In men, resection of HCA is usually recommended, irrespective of lesion size, because of the higher prevalence of malignancy [7].

However, with the evolving knowledge, especially on genetic and molecular characteristics of the disease, the treatment strategy could be also defined by the differences in risks of complications for the various adenoma subtypes [7].

The highest risk of malignant transformation into HCC is found with HCA with beta-catenin activation (b-HCA) [4]. Inflammatory HCA (I-HCA) may also exhibit marked WNT signaling pathway activation in 10% of cases, constituting a subset of tumors prone to undergo a malignant transformation, called I-HCA with beta-catenin activation (b-IHCA) [1, 4, 8, 9]. b-IHCA have features of both I-HCA and b-HCA and they carry the same risk of malignant transformation as b-HCA [10].

Stratification according to molecular subgroups at risk for complication could modify patient care [7]. A better selection of exactly those patients presenting with an HCA with an amplified risk of malignant degeneration is advocated in order to reduce the number or extension of liver resection, reducing the operative risk for these predominantly young patients and plan the most suitable routine surveillance [5].

However, because HCA can progress to malignancy, borderline hepatocellular lesions can be expected. These HCA do not have a definite pathomolecular subtype, but are examples of an uncertain diagnosis between HCA and HCC [4].

Bedossa et al. proposed the term “well-differentiated hepatocellular neoplasm of uncertain malignant potential” (HUMP) for neoplasms that cannot be confidently classified dichotomously as either HCA or carcinoma [11]. However, there are controversies if HUMP is indeed a final diagnosis or an interim statement and further studies are needed [12].

Here, we report a case of a well-differentiated hepatocellular neoplasm with atypical features in a young woman, which constituted a clinical challenge for the difficulty in determine the malignant potential and prognosis.

## 2. Case Report

The patient is a 29-year-old woman, referred to our Hepato-Bilio-Pancreatic and Splenic Unit, with a history of continuous abdominal pain in the right upper quadrants with periods of sharpening, abdominal distension, perception of a mass in the right inferior quadrant, and constipation, for the last 3 months.

She had a past history of polycystic ovary syndrome and gastritis. The patient had no children, had menarche at 14 years with regular menstrual cycles and had a history of oral contraceptive intake for 13 years. She had no history of hepatic disease, diabetes, hypertension, or dyslipidemia, and denied smoking or recreational drug usage. Presented an IMC of 16 kg/m^2^.

Her regular medication was chlordiazepoxide + clidinium bromide and the oral contraceptive estradiol valerate 1–3 mg + dienogest 2–3 mg for the last 5 years.

**Figure 1:**
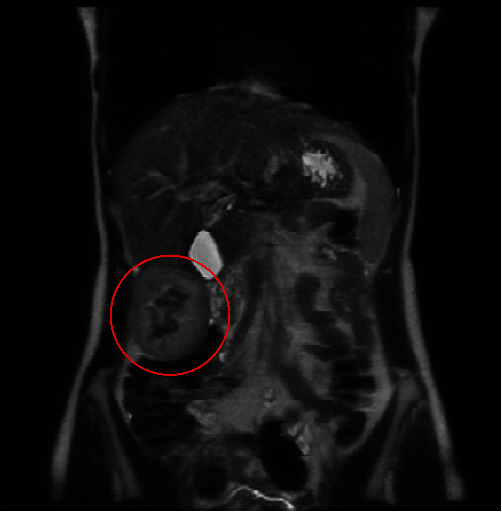
MRI showing nodular lesion arising from right hepatic lobe, in dynamic evaluation, after Gd–EOB–DTPA administration, heterogeneously captures the contrast product in a mainly peripheral way, and maintaining areas of central hyposignal.

An abdominal ultrasound showed the presence of a solid nodular lesion with origin in the right hepatic lobe. A computerized tomography scan confirmed the presence of a solid pediculate nodular lesion in segment 5. An MRI with administration of paramagnetic hepatospecific contrast product, showed an exophytic nodular lesion with 56 mm × 83 mm heterogeneous signal in the different weightings, heterogeneously capturing the contrast product in a mainly peripheral way, maintaining areas of central hyposignal probably with a more fibrosis/hyalinization component (Figure 1), in an otherwise normal liver.

Liver function tests were normal, with a slight increase in total bilirubin (BT 2.05 mg/dl). Other parameters, including leukocytes, hemoglobin, platelet counts, and renal function tests, were normal. Serum tumor markers (alphafeto-protein, CEA, and CA19-9) were normal. Serum virus markers (HBV, HCV, and HIV) were negative.

The case was discussed in our Hepato-Bilio-Pancreatic Multidisciplinary Tumor Board and it was decided surgical resection was appropriate.

A laparoscopic approach showed a pedunculated hepatic lesion arising from segment 5, not adherent to adjacent structures, without ascites, with no evidence of peritoneal carcinomatosis, or other hepatic lesions confirmed by an intraoperatively ultrasound. The resection was carried out, and the patient was discharged at day 2.

**Figure 2:**
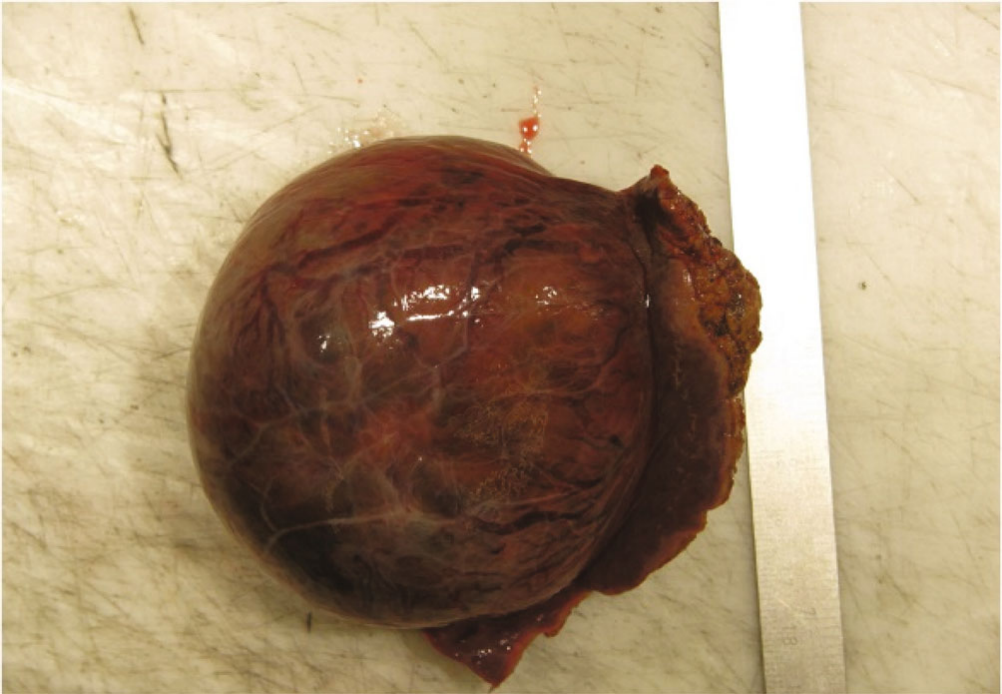
Surgical resection specimen with 8.4 cm × 7.9 cm × 5.0 cm and 192 g.

**Figure 3:**
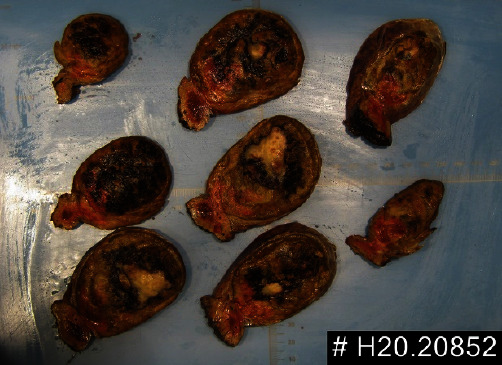
Longitudinal cut—tumor with 7.9 cm × 6.3 cm × 4.7 cm is 1.5 cm from the surgical margin.

**Figure 4:**
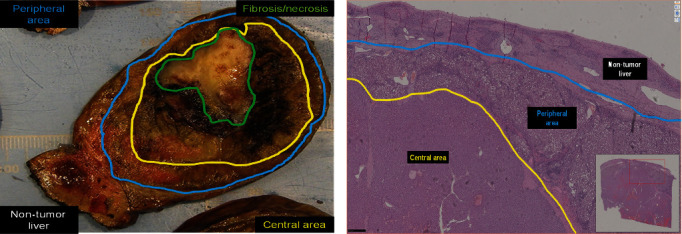
(a) The tumor is well defined with different layers and a fibronecrotic core. (b) Lower magnification of the histological features of the tumor with different layers and in the periphery non-tumor liver (H&E 40×).

The surgical specimen revealed a well-defined tumor with 7.9 cm in largest dimension (Figure 2). On the cut surface the tumor had a central fibronecrotic core and two distinct peripheral areas were apparent. The surgical margin had no neoplasia. The tumor was confined to the liver parenchyma without invasion of the hepatic capsule (Figures 3 and 4).

**Figure 5:**
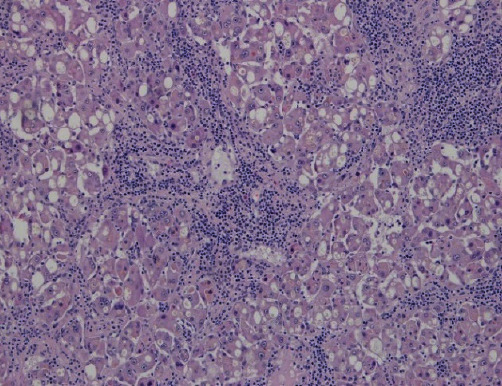
Peripheral area—H&E 100×.

**Figure 6:**
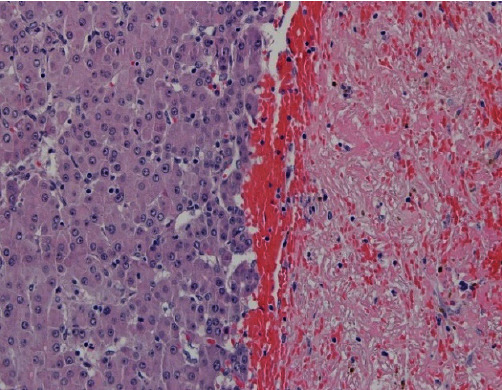
Central area—H&E 200×.

Histological examination showed a hepatocellular tumor with a peripheral area with a predominant trabecular pattern with steatosis, cholestasis, and fibrovascular septa with a lymphocytic inflammatory infiltrate (Figure 5). In the central area, there were foci of atypical cells and necrosis (Figure 6). There was no vascular or perineural invasion.

**Figure 7:**
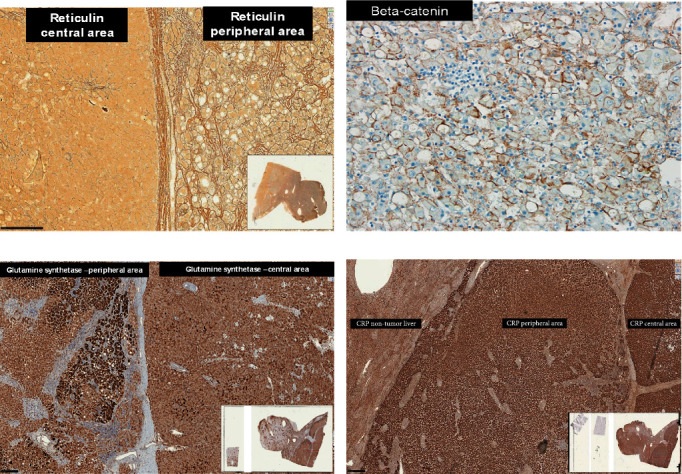
(a) Loss of reticulin fibers in the inner layer of the tumor (reticulin stain—40×). (b) Immunohistochemical study: tumoral cells with cytoplasmic expression of beta-catenin with no nuclear expression of beta-catenin (beta-catenin 100×). (c) Expression of GS (GS 40×). (d) Diffuse expression of CRP (GS 40×).

Immunohistochemical study showed expression of HEP-PAR-1, liver fatty acid-binding protein (LFABP), C-reactive protein (CRP), and glutamine synthetase (GS) in the neoplastic cells (Table 1) The beta-catenin expression was membranous only (Figure 7). The tumoral cells had no expression of glypican-3 (GPC-3) and heat-shock protein 70 (HSP-70) (Table 1).

This tumor was diagnosed as a well-differentiated hepatocellular neoplasm with morphologic characteristics of an inflammatory adenoma, probably with a beta-catenin mutation (the diffuse, but heterogeneous expression of GS and nuclear negativity for beta-catenin pointed to mutation in the exon 3 S45) with a central area with atypia, absence of biliary ducts, loss of reticulin fibers, and sinusoidal capillarization (Figures 6 and 7).

Investigation for mutations in the CTNNB1 gene (NM_001904.3; beta-catenin, the entire coding sequence including exon–intron transitions), was made through the polymerase chain reaction (PCR) technique, with direct sequencing of PCR products obtained from tumoral DNA. However, no mutations were found.

## 3. Discussion

Malignant transformation of HCA into HCC is a rare phenomenon with a reported frequency of 4–10% [5].

The distinction of HCA from well-differentiated HCC can be difficult in some cases, because of similar imaging characteristics and histopathological features, and pose significant diagnostic issues [5, 9].

The problematic lesions are neoplasms resembling HCAs, but with atypical pathological (focal cytological/architectural atypia, beta-catenin activation, or focal reticulin loss) and/or clinical features (in females over 50 years old or under 15 years, in males, with anabolic steroid use, or in some congenital conditions), which do not clearly fit within current HCA subtypes or well-established criteria for the diagnosis of HCC [13, 14].

The recognition of a category designated HUMP is helpful to identify cases that require further study so that the diagnostic criteria, including histologic features and molecular signatures, for HCA and HCC can be clarified [11].

As HCA are difficult to discriminate from HCC, it is important to have markers to identify high-risk adenomas [5].

The immunohistochemical markers GS and nuclear beta-catenin characterize the b-HCA subtype that carry a high risk of malignant transformation [5, 8]. HCC associated with an adenoma is found in up to 46% of beta-catenin-mutated tumors [5, 15].

It should be noted that b-HCA is a heterogeneous group of tumors with various levels of activation of the WNT signaling pathway, as a result of mutations or deletions of the CTNNB1 gene involving exons 3, 7, and 8. Of the three subtypes, the one with exon 3 abnormalities (except S45) has the highest risk for malignant transformation into HCC [4].

Large deletions and most hotspot mutations in exon 3 lead to high levels of beta-catenin activation, whereas exon 3 S45 and exon 7/8 mutations lead to moderate and weak activation of the pathway, respectively, leading to different GS patterns. Diffuse homogeneous GS staining is observed with mutations that lead to strong beta-catenin activation, whereas more-modest activation tends to show a diffuse heterogeneous pattern. Therefore, GS is a good surrogate marker to identify different types of CTNNB1 mutations [10].

In a retrospective multicentric study, the predictive potential of three known GS patterns as markers for CTNNB1 exon 3, 7/8 mutations were investigated. Pattern 1 (diffuse homogenous) allowed recognition of 17/21 exon 3 non-S45 mutated b-(I)HCA. Pattern 2 (diffuse heterogenous) identified all b-IHCA harboring exon 3 S45 mutation (20/20). Pattern 3 (focal patchy) distinguished 12/22 b-IHCA with exon 7/8 mutations. In exon 3 S45 and 7/8 mutations, both b-HCA and b-IHCA showed a GS+/CD34− rim with diffuse CD34 positivity in the center of the lesion [16].

Importantly, nuclear expression of beta-catenin is not a sensitive marker of beta-catenin activation and is seen in less than half of cases. In addition, diffuse GS staining may not necessarily signify beta-catenin activation, the reported correlation between diffuse GS staining and CTTNB1 (beta-catenin) mutations ranges from 15 to 100% in HCC and 75–100% in HUMP. Despite the lack of complete correlation with beta-catenin activation, diffuse GS staining is currently considered a high-risk feature [9].

In the immunohistochemical study of the presented case diffuse expression of GS and membranous immunohistochemical expression of beta-catenin, but not nuclear expression was noted.

The concordance between nuclear beta-catenin staining and diffuse GS staining is high, but some tumors show diffuse and strong GS expression in the absence of nuclear beta-catenin staining. This phenomenon has been observed in HCA-like area in 29–44% of cases. The reason for the discrepancy between GS and beta-catenin expression is not known, some of these cases show beta-catenin mutation without nuclear beta-catenin or may have mutations affecting other components of the WNT signaling pathway, such as AXIN1 and AXIN2 [6]. Accordingly, although the presented case showed characteristics that pointed to beta-catenin mutation, it was not proven by genetic analysis. It is possible there may be an activation on the WNT/beta-catenin pathway by another gene mutation.

Other immunohistochemical biomarkers of malignancy can play an important role in identifying high-risk hepatocellular neoplasms, such as GPC-3 and HSP-70, and these may be useful tools to support the diagnosis of HCC [1, 9].

In a study comparing gene expression between HCC and other hepatocellular nodules, HSP-70 was the most discriminatory gene. However, there is limited information about HSP-70 staining in HCA, with numbers ranging from 0 to 40% in three studies, further strengthening the argument that these areas may be extremely well-differentiated HCC [6].

HSP-70, GS, and GPC-3 are usually negative in typical HCA, whereas positive staining with HSP-70 and/or GPC-3 would point towards HCC [9]. In our case expression of GPC-3 and HSP-70 was absent.

The differentiation between HCA with benign or malignant characteristics is important to define the treatment and surveillance strategy, because of the risk of disease progression. For this reason, a case of HCA with atypical characteristics raises doubts and difficulties about the nature of the tumor, with treatment implications.

In this case the label of a HUMP tumor was initially considered with a central foci of atypical cells, with diffuse heterogeneous expression of GS, but nuclear negativity for beta-catenin and no mutations found in the CTNNB1 gene, and expression of GPC-3 and HSP-70 was absent. However, extensive loss of reticulin fibers, considered greater than the “focal” loss described by the HUMP criteria, and diffuse CD34 positivity in the central area suggested instead that this was malignant transformation in a well-differentiated HCC.

HCC developing from HCA is thought to behave less aggressively and the long-term prognosis for patients with HCA with malignant transformation who undergo resection is generally good, and recurrence is uncommon [17]. However, some patients have disease progression with local recurrence and/or extrahepatic metastasis [18].

In a single-center experience of 122 patients with HCA, all patients treated by resection survived, without recurrent malignancy, and after a mean follow-up period of 78 months. 109 patients with benign HCA revealed recurrence or progression in 8% and regression in 9% of cases with residual HCA. Results of this study showed that complete resection of malignant HCA is a safe and efficient option because none of the patients experienced malignant recurrence over a mean follow-up period of 7 years [17]. Analysis of malignant HCA showed that HCC is well differentiated and developed on large HCA (>8 cm) in all cases, except in one male with a history of anabolic steroids intake. Malignant HCA was not associated with vascular involvement or lymph node metastasis in any of the cases. Among the 10 patients with malignant HCA, abnormal beta-catenin staining (suggesting mutations), was observed in 2 (20%) cases [17].

A study [19] with 118 patients with a median follow-up of 5 years, showed that 78% of HCA had long-term stability or regression (90% in solitary HCA and 71% in multiple HCA). After resection of solitary HCA, new lesions occurred in 7%, only in HCA at risk of progression (b-HCA and HCA with foci of malignancy) [19].

Accordingly, MRI follow-up may be discontinued in patients with a solitary HCA after resection unless worrisome features are detected at pathologic assessment. Patients with HCA at risk of progression or multiple HCA should undergo continuous follow-up regardless of surgery [19].

After multidisciplinary discussion, this case was considered a well-differentiated HCC and was decided a continuous surveillance. At 2-year follow-up, the patient remains asymptomatic with no imaging evidence of recurrence.

However, what about lesions that do not meet the threshold for resection (<5 cm). It is known that beta-catenin mutated HCA has higher risk to progress to HCC. Subtyping of HCA has become increasingly important clinically, because HCAs that are more highly associated with malignant progression are more likely to be resected or ablated, whereas those that are not are more likely to be followed with imaging. Although subtyping is fairly widely available and is often requested by clinicians, the most recent American and European clinical guidelines do not require subtyping for routine practice [20].

Would it be important to stratify according with the HCA subtype to better establish the treatment in lesions that do not have clear criteria to resection, proceeding with ultrasound-guided percutaneous fine needle aspiration (FNA), endoscopic liver biopsy, or even laparoscopic biopsy?

Ultrasound-guided percutaneous FNA and endoscopic liver biopsy procedures are safe, low-cost, efficient, and minimally invasive procedures that have high sensitivity, specificity, and accuracy rate when diagnosing mass lesions of the liver [21, 22]. In a retrospective study looking at over 4,000 cases, including primary benign and malignant liver lesions and metastatic lesions, the sensitivity of FNA was 97%, with specificity of 100%, and an accuracy rate of 71% in primary liver lesions [21]. In another large study, the positive and negative predictive values and overall accuracy of FNA diagnosis for liver malignancy were reported to be 100%, 59.1%, and 92.4%, respectively. False positives are rare [22].

However, may be challenging to even unequivocally diagnose focal nodular hyperplasias, HCA, regenerative nodules, and well-differentiated HCC by FNA and the role and efficacy of FNA in small hepatocellular nodular lesions (less than and equal to 2 cm) is actively debated. On FNA and cell block material, these lesions are characterized by hepatocytes without significant atypia and trabeculae that are two cells thick. The diagnosis of HCA can only be made on needle core biopsies, because they are characterized by absent portal tracts and preserved hepatic trabeculae thickness, which are highlighted by the reticulin stain. In addition, in case of HCA, ancillary stains, including immunohistochemical stains, reticulin stain, and GPC-3 are required in addition to hematoxylin and eosin-stained sections, to subtype beta-catenin mutated adenomas [20–22].

Additionally, FNA biopsy is not without its complications, albeit rare. They include intraperitoneal bleeding, needle tract seeding, and alleged intraprocedural hematogenous dissemination with tumor recurrence, and the rare fatality. A mortality rate of 0.018% was reported in a multi-institutional Italian series of 10,766 ultrasound-guided FNA biopsies [22].

All these reasons have contributed to reluctance in obtaining routine pre-operative FNA diagnosis. The conundrum is to balance if the biopsy is really necessary and if it does change the outcome, against the risk of the procedure. If resection appears to be the best option, biopsy may not be performed [22].

## 4. Conclusion

HCA share similar imaging characteristics and histopathological features with HCC; therefore, immunohistochemical and genetic studies assumes great importance to discriminate adenomas with malignant transformation. Beta-catenin, GPC-3, and HSP-70 are promising markers to identify higher risk adenomas.

The prognosis for those afflicted with HCA is not predictable, and it remains clinically challenging to manage and counsel these patients.

Because of the higher risk of malignant transformation of HCA, resection is recommended in men, patients with beta-catenin exon 3 mutations (except S45) irrespective of tumor size, large HCA (≥5 cm), and in borderline lesions.

HCA is a rare entity; therefore, all cases must be discussed at multidisciplinary liver tumor boards that include specialized radiologists, pathologists, hepatologists, and surgeons to determine the most suitable patient-oriented treatment strategy.

## Figures and Tables

**Table 1:** Immunohistochemical study of the tumor.

	Peripheral area	Central area
CD34		
Clone QBEnd-10, Leica, 1 : 100 dilution	±	+++ (Sinusoidal capillarization pattern)
GS		
Clone 6, BD Transduction Laboratories™, 1 : 600 dilution	+ (Heterogeneous)	++ (Diffuse)
SAA		
Clone mc1, Dako, 1 : 100 dilution	Inconclusive	Inconclusive
CRP		
Clone y284, Abcam, 1 : 500	+++	+++
LFABP		
Clone EPR20464, Abcam, 1 : 1,000	Preserved in neoplastic cells	Preserved in neoplastic cells
Glypican-3		
Clone GC33, Ventana Medical Systems, pre-diluted	−	−
HSP-70		
Clone W27, NeoMarkers, 1 : 100	−	−
Beta-catenin		
Clone 14, Cell Marque, pre-diluted	+ (Membranar)	+ (Membranar)
CK7		
Clone SP52, Ventana Medical Systems, pre-diluted	+ (Biliary ducts)	− (Absence of biliary ducts)
CK19		
Clone A53-B/A2.26, Cell Marque, pre-diluted		

## Data Availability

Data sharing is not applicable for this article as no datasets were presented or analyzed during the current study.
